# The complex nature of CXCR4 mutations in WHIM syndrome

**DOI:** 10.3389/fimmu.2024.1406532

**Published:** 2024-07-05

**Authors:** José Miguel Rodríguez-Frade, Luis Ignacio González-Granado, César A. Santiago, Mario Mellado

**Affiliations:** ^1^ Department of Immunology and Oncology, Chemokine Signaling Group, Centro Nacional de Biotecnología/CSIC, Madrid, Spain; ^2^ Department of Pediatrics, 12 de Octubre Health Research Institute (imas12), Madrid, Spain; ^3^ Department of Public Health School of Medicine, School of Medicine, Universidad Complutense de Madrid, Madrid, Spain; ^4^ X-ray Crystallography Unit, Centro Nacional de Biotecnología/Consejo Superior de Investigaciones Científicas (CSIC), Madrid, Spain

**Keywords:** CXCR4, CXCL12, WHIM syndrome, receptor conformations, signaling pathways, β-arrestins

## Abstract

Heterozygous autosomal dominant mutations in the CXCR4 gene cause WHIM syndrome, a severe combined immunodeficiency disorder. The mutations primarily affect the C-terminal region of the CXCR4 chemokine receptor, specifically several potential phosphorylation sites critical for agonist (CXCL12)-mediated receptor internalization and desensitization. Mutant receptors have a prolonged residence time on the cell surface, leading to hyperactive signaling that is responsible for some of the symptoms of WHIM syndrome. Recent studies have shown that the situation is more complex than originally thought, as mutant WHIM receptors and CXCR4 exhibit different dynamics at the cell membrane, which also influences their respective cellular functions. This review examines the functional mechanisms of CXCR4 and the impact of WHIM mutations in both physiological and pathological conditions.

## CXCR4: a key player in health and disease

The elucidation of the CXCR4 gene and its impact on human immunology followed several key discoveries in the 1990s and the early 2000s. The CXCR4 gene was first identified and characterized in the 1990s. Initial efforts mapped the gene to chromosome 2q21 using isotopic *in situ* hybridization (1, 2). The genomic structure of the CXCR4 gene was determined by Wegner et al. in 1998 (3). Key elements in the CXCR4 promoter include a TATA box, a nuclear respiratory factor-1 (NRF-1) site, and two GC boxes, which are critical for the regulation of gene expression.

CXCR4 is a unique polypeptide organized into seven transmembrane α-helices that interact with heterotrimeric G proteins to activate intracellular signaling pathways. Originally described as an orphan G protein-coupled receptor (GPCR), CXCR4 was later found to facilitate HIV-1 fusion with target cells (4). CXCR4 is a homeostatic receptor that is widely expressed in both embryonic and adult tissues (5) and has a unique chemokine ligand, CXCL12 (6), although it can also bind other ligands including CXCL14 (7), migration inhibitory factor (MIF) (8) and HMGB1, which forms complexes with CXCL12 (9). Studies in mice deficient in CXCR4 (*Cxcr4^-/-^*) have demonstrated significant impairments in hematopoiesis, and in the development of the nervous and cardiovascular systems. The importance of the CXCR4/CXCL12 axis is underscored not only by the embryonic lethality of both CXCR4- and CXCL12-deficient mice, but also by its high degree of conservation throughout evolution (10).

The CXCR4 protein was first crystallized in 2010 and five independent crystal structures of CXCR4 bound to a small molecule antagonist and a cyclic peptide were reported (11). These studies revealed a consistent homodimer interface involving helices V and VI. Recently, a comparative structural and phylogenetic analysis of CXCR4 sequences from 30 mammalian species revealed a complex evolutionary history with multiple gene duplication and loss events, along with the identification of key functional domains and amino acid residues conserved across species (12).

Similar to other chemokine receptors, CXCR4 plays a pivotal role in leukocyte trafficking and arrest in specific anatomical niches under both homeostatic and pathological conditions (13). Indeed, the CXCL12/CXCR4 axis is essential for both adaptive and innate immune responses, as well as for the organization and maintenance of the bone marrow (BM) (14). Importantly, CXCR4 is critical for the migration, homing and survival of hematopoietic stem cells (HSC) in the BM (15–18). CXCL12 is primarily produced by perivascular mesenchymal stem cells and is essential for HSC quiescence and retention in the BM (19). During the establishment of antigen-presenting cell-T cell contacts, CXCR4, together with other chemokine receptors, is recruited to the peripheral supramolecular activation cluster where it contributes to integrin activation, necessary for the formation of a productive immunological synapse and correct T cell activation (20). Furthermore, B cell organization in the germinal centers of lymph nodes is dependent on CXCR4 expression, and CXCR4-deficient B cells are excluded from the germinal center dark zone (21).

CXCR4 is also expressed in non-hematopoietic tissues including lung, liver, kidney, gastrointestinal tract, adrenal gland, ovary and brain. Conditional *Cxcr4* ^-/-^ mouse models have demonstrated the importance of CXCR4 in regulating the development of the central nervous system (22), as well as the development of the vasculature in the gastrointestinal tract (23) and kidney (24).

Importantly, CXCR4 and CCR5, another chemokine receptor, serve with CD4 as primary co-receptors for HIV-1 entry into target cells (25). Data suggest that viral use of CXCR4 correlates with immunodeficiency and progression to AIDS (26, 27).

Although CXCR4 is primarily considered a homeostatic receptor, its expression can be modulated in various pathological situations. For example, CXCR4 is overexpressed in many tumor types, including breast (28), lung (29) ovarian (30), prostate (31), colon (32), melanoma (33) and neuroblastoma (34). Increased CXCR4 expression in metastatic lesions correlates with tumor progression and with preferential metastatic sites of the primary tumor (28, 35, 36). Studies in mice have shown that CXCR4 blockade inhibits cancer cell dissemination and metastasis in several cancer models (37, 38). The CXCL12/CXCR4 axis is also involved in tumor growth, tumor cell-microenvironment interactions (39), vasculogenesis and angiogenesis (40).

Inflammation is another mechanism involved in the modulation of CXCR4. Transforming growth factor-beta 1 (TGF-β1) (41), vascular endothelial growth factor (VEGF) (42) and basic fibroblast growth factor (bFGF) (43) have all been reported to upregulate CXCR4 expression, whereas cytokines such as IL-5 (44), interferon-alpha (IFN-α) and interferon-gamma (IFN-γ) (45) have the opposite effect. In addition, activation of CXCR4 in macrophages after LPS stimulation suppresses the expression of inflammatory cytokines by blocking MAPK and NF-kB signaling pathways (46). Taken together, these findings support the involvement of CXCR4 in the development and progression of immunodeficiency, inflammatory diseases and cancer and highlight its potential as a therapeutic target.

## Altered immune function in patients with WHIM syndrome

Heterozygous autosomal dominant mutations in the CXCR4 gene cause WHIM syndrome (47, 48) a severe combined immunodeficiency disorder characterized by increased susceptibility to human papillomavirus pathogenesis, resulting in warts, condyloma acuminata and carcinomas. Patients with WHIM syndrome often present with neutropenia, a common symptom in several primary immunodeficiencies (**Table 1**) (49, 50), B cell lymphopenia, hypogammaglobulinemia, recurrent infections and myelokathexis characterized by myeloid hyperplasia and an overabundance of mature senescent neutrophils in the BM (48). Some patients also have developmental defects of the cardiovascular, genitourinary and nervous systems (51), although only the cardiovascular defects appear to be clinically significant (52–54). Moreover, decreases in bone mineral density and bone defects leading to osteoporosis have been reported in a WHIM mouse model (55). Some defects in T cell activation in WHIM syndrome may also be associated with a compromised stability of the immunological synapse formed during T cell-APC engagement (56, 57). While CXCR4 gain-of-function variants are the most common cause of WHIM syndrome, a proportion of patients remain undiagnosed. While some patients harboring CXCR2 mutations show myelokathexis or neutropenia, the absence of other features of WHIM syndrome, indicate that this CXCR2 deficiencies have characteristics distinct from those of WHIM (58, 59).

**Table 1:** Differential diagnosis of WHIM syndrome.

NON-SYNDROMIC NEUTROPENIA	OCULOCUTANEOUS ALBINISM	EXOCRINE PANCREATIC INSUFFICIENCY	COMBINED IMMUNODEFICIENCY	BONE MARROW FAILURE	OTHER SYNDROMIC NEUTROPENIA
SEVERE CONGENITAL NEUTROPENIA AD: ELANE, GF11	CHS HPS2 GS2	SCHWACHMAN DIAMOND SYNDROME (SBDS/DNAJC21/EFL1)	WHIM/CXCR2	CONGENITAL DISKERATOSIS	G6PC3
AR: HAX1, JAGN1, CSF3R, G6PC3, WAS, VPS45A	P14/LAMTOR2	SRP54	CD40LG	GATA2	COHEN SYNDROME (VPS13B)
XL: N-WASP, XLA			RETICULAR DISGENESIS (AK2)	FANCONI ANEMIA	BARTH SYNDROME (TAZ)
			MOESIN		
			EZRIN	IRF8	POIKILODERMA
			CARD11		

Type of monogenic neutropenias by subgroup. AD, Autosomal Dominant; AR, Autosomal Recessive; XL, X- Linked; CHS, Chediak-Higashi Syndrome; HPS2, Hermansky-Pudlak Syndrome type 2; GS2, Griscelli Syndrome type 2; SRP54, Signal Recognition Particle 54; CD40LG, CD40 Ligand Deficiency (Hyper-IgM Syndrome); CXCR2, C-X-C Motif Chemokine Receptor 2; G6PC3, Glucose-6-Phosphatase Catalytic Subunit 3; GATA2, GATA Binding Protein 2; P14/LAMTOR2, Late Endosomal/Lysosomal Adaptor, MAPK and MTOR Activator 2; AK2, Adenylate Kinase 2; N-WASP, Neural Wiskott-Aldrich Syndrome Protein; XLA, X-linked Agammaglobulinemia; MOESIN, Membrane-Organizing Extension Spike Protein; SAMD9/SAMD9L, Sterile Alpha Motif Domain-containing 9/9-like; GSD1b, Glycogen Storage Disease type 1b (SLC37A4); EZRIN, Ezrin Protein; IRF8, Interferon Regulatory Factor 8; USB1, U6 SnRNA Biogenesis Phosphodiesterase 1; SMARCD2, SWI/SNF Related, Matrix Associated, Actin Dependent Regulator of Chromatin Subfamily D Member 2.

Modified from Spoor et al. and Hauck and Klein (49, 50).

Little is known about the contribution of WHIM mutations to HIV-1 infection, with the exception of one report claiming similar or even lower susceptibility to infection (48) or about the role of WHIM expression in inflammation (60). Paradoxically, WHIM syndrome behaves as a relatively benign immunodeficiency. Indeed, the majority of infections are usually not invasive or life-threatening, and patients survive into adulthood, in part due to the ability of the host response to acute infections to mobilize leukocytes into the circulation. Patients with WHIM do not show a clear genotype-phenotype correlation. In fact, there is considerable phenotypic variability between patients with the same genotype, even within the same family (61, 62). However, they have several unmet needs, such as long-term immunoglobulin replacement therapy and the management of periodontal disease, which affects up to 63.6% of patients with moderate or severe periodontitis (63).

At the molecular level, WHIM syndrome is caused by heterozygous gain-of-function mutations at the C-terminal end of CXCR4 that affect key residues involved in receptor phosphorylation and desensitization (64), explaining the associated hyperactivation of downstream signaling and the retention of leukocytes in the BM, causing robust neutropenia (65). Consistent with this, mice with a myeloid lineage-restricted deletion of CXCR4 also exhibit marked neutrophilia (66). These data indicate that CXCR4 has a dual role in neutrophil homeostasis, regulating both neutrophil release from BM and clearance from blood. CXCR4 expression increases in senescent neutrophils and blocking anti-CXCR4 antibodies abolish neutrophil homing to BM (67, 68). In addition, the p.H323fs329X mutation in CXCR2, the receptor involved in neutrophil egress from BM, is also associated with a similar reduction in circulating neutrophils (58, 67). The severe neutropenia, triggered by delayed neutrophil egress into the circulation and enhanced neutrophil homing to BM, determines the known susceptibility to bacterial and viral infections associated with these patients. Several of the mutations described in WHIM syndrome are also found in patients with Waldenström’s macroglobulinemia, a rare B-cell lymphoma (69) characterized by lymphoplasmacytic infiltrates in the BM, lymph nodes and spleen, often associated with the presence of high IgM titers in the blood (70).

For decades, G-CSF was the only drug available to increase neutrophil and lymphocyte counts in patients with WHIM syndrome. Initial results from phase 1 trials of plerixafor, a selective CXCR4 inhibitor, were reported in 2014 and 2019 in open-label studies (71). Recently, results of a phase 3 crossover randomized controlled trials (RCT) of 19 patients treated with either plerixafor or G-CSF over 12 months became available. While plerixafor was not superior to G-CSF in reducing the overall infection severity score (the primary endpoint), it did result in wart regression and hematological improvement (72).

By contrast, patients with WHIM syndrome participating in a phase 2 trial of mavorixafor (400 mg *quaque die*, administered orally, a more convenient option than subcutaneous infusion) not only had increased total white blood cell, neutrophil and lymphocyte counts, but also showed reduced annualized infection rates and reduced wart numbers in those treated for six months or more. Results from the phase 3 trial confirmed positive outcomes in terms of the primary endpoints: time (hours) above the absolute neutrophil count (ANC) threshold ≥500/μL, time (hours) above the lymphocyte threshold, and reduced infection frequency, severity and duration (73).

A functional cure of WHIM syndrome has been reported in a patient following chromothriptic deletion of the abnormal CXCR4 gene in hematopoietic stem cells, suggesting the potential of gene editing as a future therapeutic approach (74). Novel CRISPR-Cas9 base editing techniques, including cytosine base editors (CBEs) and adenine base editors (ABEs), along with prime editing, might offer curative treatment without the known risks of hematopoietic stem cell transplantation (HSCT). In a global cohort of 66 patients, the only patient who died had undergone HSCT. Notably, the CRISPR-Cas9 approach successfully corrected the disease in a WHIM mouse model (75).

## WHIM mutations shape CXCR4 downstream signaling

CXCR4 signaling is finely coordinated by physical receptor interactions with multiple proteins (G proteins, G protein receptor kinases [GRKs] and β-arrestins, filamin A, cofilin, etc) (76, 77). CXCL12 binding to CXCR4 triggers Gα_i_ activation, although signaling involving Gα_12-13_ and Gα_q_ has also been described (78, 79). One report has linked the induction of Gα_q_ by the HIV-1 envelope glycoprotein through CXCR4 to viral entry (80). These initial signaling events trigger the activation of multiple signaling pathways, including those associated with Src, PI3K, PLC, PKC and MAPK (81).

Binding of CXCL12 to CXCR4 triggers rapid phosphorylation within its 45 amino acid serine/threonine (Ser/Thr)-rich region, primarily at the distal C-terminus. Phosphorylation is critical for agonist (CXCL12)-mediated receptor internalization (82, 83) and degradation (84) and depends on the activity of GRK2 (85, 86), GRK3 (87) and GRK6 (88). GRK-mediated phosphorylation of residues along the C-terminus of CXCR4 facilitates the recruitment and activation of β-arrestins, which mediate receptor desensitization and internalization. It has also been reported that CXCR4 and other chemokine receptors show constitutive activity and internalization in the absence of β-arrestins (89–91), an effect that is attributed to PKC (82). In addition, β-arrestins have a scaffolding role (92) linking the activated receptor to the actin cytoskeleton via several actin-binding proteins (i.e., FLNA and cofilin) (93, 94). This allows the transmission of the CXCL12-mediated conformational changes in CXCR4 to drive signal transduction that triggers the cytoskeletal rearrangements required for cell polarization and the formation of a leading edge, and to sustain productive chemokine-directed cell migration (95). These observations are also consistent with the stabilization of different β-arrestin conformations on the receptor depending on the phosphorylation residues present in the C-tail of the receptors (96).

**Figure 1:**
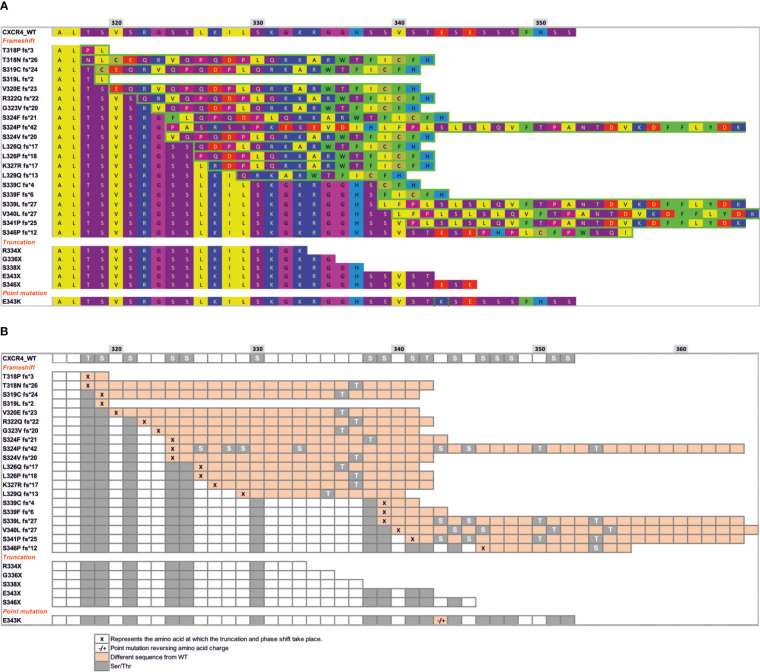
Mutations at the C-terminal end of CXCR4 WHIM receptors lead to different potential changes in the Ser/Thr phosphorylation pattern. **(A)** Sequence of CXCR4 residues (318–352) showing the three different types of mutations that occur in WHIM syndrome: frameshift, truncation or nonsense and missense point mutations. Hydrophobic residues are shown in yellow, bulky hydrophobic residues in green, hydrogen donors in purple, and positively and negatively charged residues in blue and red, respectively. Residues framed in green show a different sequence from wild-type CXCR4, resulting from a frameshift or point mutation. **(B)** Scheme showing the pattern of phosphorylation sites present in helix 8 and in the c-terminal region of CXCR4, shown as grey colored squares. Changes in the sequence following a frameshift or point mutation (-/+) are shown in orange boxes following the sequence from **(A)**. Specific residues where the frameshift occurs are shown as ‘x’ in orange boxes.

**Figure 2:**
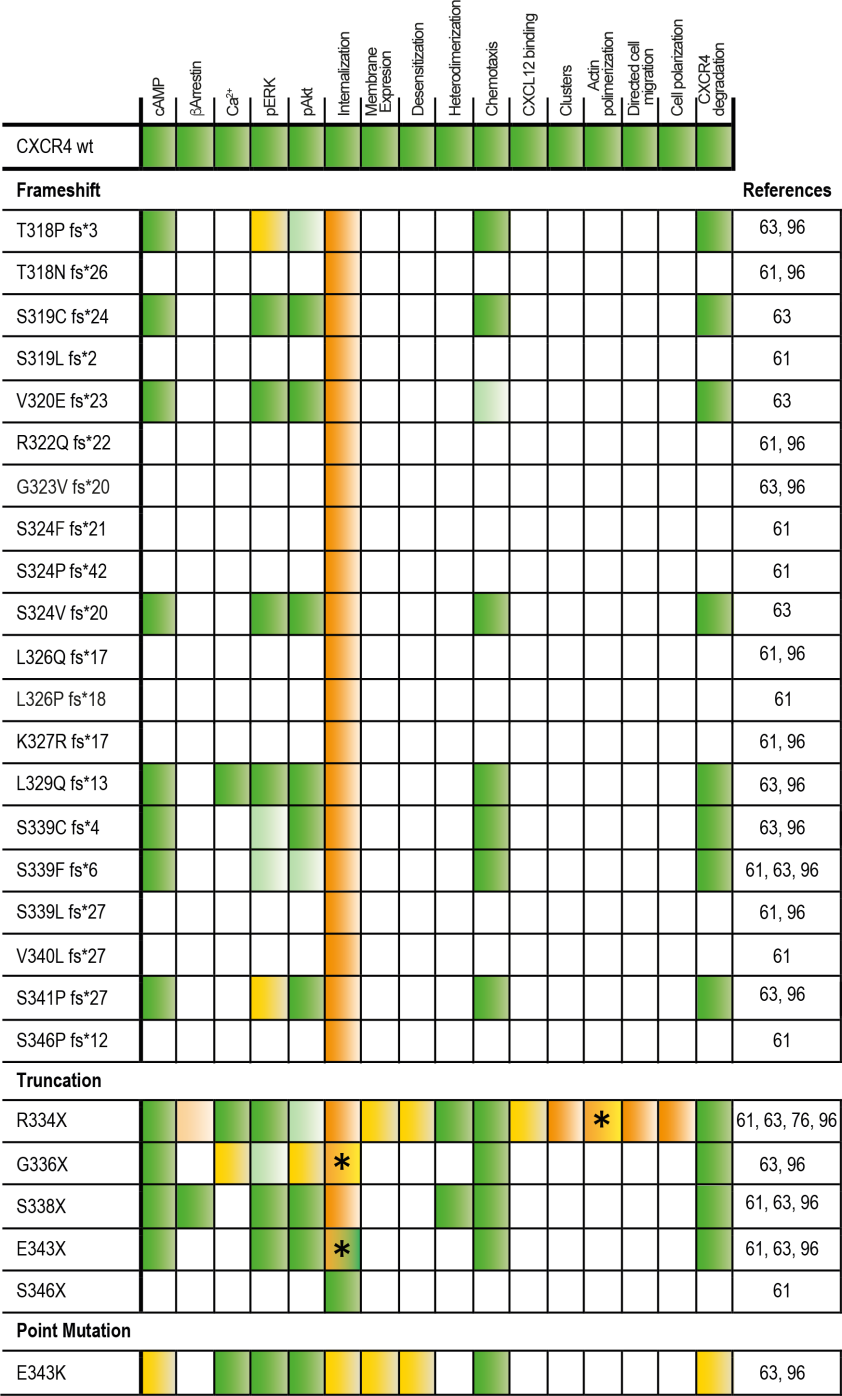
Response to CXCL12 of the different WHIM mutants as compared with the wild-type receptor. An increase in the response to CXCL12 compared with the wild-type receptor is indicated by green boxes (light green indicates a weak but significant response). A decrease in the response is indicated by red boxes (light red indicates a weak but significant decrease). No effect of the mutation on CXCL12 responses is shown in yellow. White boxes indicate missing information. Different results depending on the concentration or the cell line used in the study are indicated by an asterisk (_*_).

Previous studies have identified 26 autosomal dominant mutations in CXCR4 associated with WHIM syndrome (64, 97). Most of these mutations affect the C-terminal receptor tail and are unlikely to affect the rest of the quaternary structure, indicating the importance of the C-terminal region in CXCR4 signaling (**Figure 1**). It is therefore not surprising that GRK-mediated phosphorylation of Ser/Thr residues, β−arrestin coupling and receptor desensitization and internalization are affected by C-terminal mutations. Indeed, WHIM mutants show impaired receptor internalization and degradation, resulting in prolonged receptor residence time at the cell membrane, which in turn contributes to the gain-of-function properties of these receptors and their hyperactive signaling nature compared with wild-type CXCR4 (**Figure 2**). Further investigation is, however, required to explore the full impact of WHIM mutations on G protein recruitment and activation (97). *In vitro* assays have shown that CXCL12-induced ^35^S-GTPγS binding to activated Gα_i_-containing membranes from cells expressing comparable levels of wild-type CXCR4, CXCR4^S338X^ or CXCR4^R334X^, the most common mutations observed in WHIM patients, results in increased coupling efficiency and potency of the mutant receptors (98–100). In addition to the defects in β-arrestin coupling, the stronger association of G proteins to mutant receptors might also contribute to the heightened responsiveness of WHIM mutant CXCR4 to CXCL12.

The most common mutation in WHIM syndrome is a truncation at Arg334 (R334X) (101) which, similar to the CXCR4^S338X^ truncation, promotes increased CXCL12-mediated signaling, G protein interactions and ERK and AKT activation, and decreased GRK6 associations, β-arrestin2 interactions and impaired receptor internalization (98–105). Both mutants are also associated with prolonged CXCL12-mediated F-actin polymerization (99). Similarly, the E343K point mutation results in increased receptor signaling, decreased Ser/Thr phosphorylation and impaired receptor internalization (106, 107), although receptor internalization was only affected at low CXCL12 concentrations (63). The case of CXCR4^E343K^ is quite interesting, as this receptor has a full length C-terminus with complete phosphorylation sites and no change in all potential phosphorylation sites. However, the negative charge of the 343 site at the receptor tail is essential for CXCR4 function, and changing the charge through an amino acid substitution dysregulates the signaling events downstream of G proteins. Cells overexpressing CXCR4^E343D^ have no functional changes compared with wild-type CXCR4, whereas cells overexpressing CXCR4^E343K^, CXCR4^E343R^ or CXCR4^E343A^ show increased cell migration, prolonged phosphorylation of ERK1/2, p38, JNK1/2/3 and increased activation of PI3K/AKT/NF-κB signal pathway, that is, all these mutants reproduce the *in vitro* signaling events associated with WHIM syndrome (108). By contrast, the CXCR4^E343X^ and CXCR4^S341P fs*25^ mutants are partially internalized at high doses of CXCL12 (63). Taken together, these data suggest that the mutant CXCR4-dependent changes in signaling may reflect differences in the symptoms experienced by patients, or may even be involved in the differential penetrance of this syndrome between patients. Defects in receptor internalization show a strong correlation with the severity of neutropenia and lymphopenia in patients with WHIM syndrome, as well as with their susceptibility to recurrent infections (64). Some studies also associate AKT hyperactivation with reduced IgA levels in blood and decreased T cell counts (64), a finding common in activated PI3K delta syndrome, which is characterized by constitutively active AKT signaling (109), and a reduction in T cell numbers has been associated with strong inhibition of cAMP release (64). These are, nonetheless, expected consequences, as these CXCR4 variants are gain-off-function receptors due to their lack of internalization.

Interestingly, some CXCR4 mutations associated with the WHIM phenotype are not consistent with a gain-of-function phenotype. For example, CXCR4^T318P fs*3^ mutants show impaired β-arrestin recruitment with decreased ERK1/2 phosphorylation and calcium mobilization, resulting in reduced cell migration in response to CXCL12 (110), whereas CXCR4^E343K^ and CXCR4^G336X^ mutants do not affect ligand-mediated internalization (97). CXCR4^E343K^ has a full-length C-terminus and the same number of Ser/Thr phosphorylation sites as wild-type CXCR4, but still functions as a gain-of-function mutant (108). CXCR4^S338X^ retains its interaction with β-arrestins, but is not internalized and induces stronger ERK phosphorylation and cell chemotaxis than wild-type CXCR4 (97). The S339F fs*6 mutation enhances agonist-driven signaling, decreases Ser/Thr phosphorylation, β-arrestin binding and endocytosis and increases basal degradation (111). While some mutations have been clearly linked to defects in the phosphorylation of specific residues required for efficient receptor endocytosis and degradation, others appear to be less essential, supporting the involvement of additional factors to explain the phenotype associated with this syndrome.

During the review process of this manuscript a a novel heterozygous CXCR4 variant (c.250G>C; D84H) localized at a highly conserved position in the transmembrane domain of the receptor outside the C-terminus has been described (112). The patient-derived peripheral blood mononuclear cells carrying this mutation, and *in vitro* cellular assays show decreased CXCR4 internalization and increased chemotaxis in response to CXCL12, similar to known CXCR4WHIM, but also revealed unique features of CXCR4D84H signaling as shows impaired cAMP inhibition and Ca2^+^ mobilization and does not show enhancement in pAKT or pERK levels as the other WHIM variants do. These findings are consistent with molecular dynamics simulations that show disruption of the Na^+^ binding pocket by D84H, resulting in collapse of the hydrophobic gate above and destabilization of the inactive state of CXCR4.

Collectively, these data suggest that WHIM syndrome is molecularly more complex than originally thought, as different mutations on receptors and different effects on CXCL12-mediated functions can lead to similar cellular phenotypes.

## Receptor dynamics add complexity to the chemokine receptors

Until relatively recently, GPCRs were thought to be monomeric entities that transiently interact with a G protein, promoting its dissociation into Gα and Gβγ subunits. Biophysical and biochemical studies of rhodopsin, the first purified GPCR, supported the concept that GPCRs are monomeric (113). Accordingly, a single activated receptor was thought to sequentially activate multiple G proteins in a simple ternary model of ligand/receptor/G protein complexes, which was considered sufficient to explain the functions triggered by ligand binding under equilibrium conditions (114). The model included two receptor populations: an inactive receptor and an active receptor. Ligand binding and G proteins cooperatively promoted the transition to the active form, initiating downstream signaling (115). In addition, both rhodopsin and β2 adrenergic receptors remained functional when entrapped as monomers in nanolipid disks, and were able to bind their corresponding ligands and activate G proteins (113, 116).

Over the past decade, however, structural and spectroscopic studies have revealed that GPCRs occupy a continuum of conformational states that progressively facilitate G protein activation (117, 118). The receptor is maintained in an inactive conformation by interhelical ionic locks that act as molecular switches, and ligand binding increase the conformational heterogeneity of the receptors, flipping these molecular switches to facilitate receptor activation (119). This is also true for CXCR4, where CXCL12 binding induces conformational changes in the transmembrane domains. A two-step binding site model has been proposed for the interaction between CXCL12 and CXCR4 (120, 121). In the first step, the central body of the chemokine interacts with the N-terminal end of CXCR4 in the chemokine recognition site 1 (CRS1), allowing optimal orientation of CXCL12 on the receptor. This allows the N-terminus to enter the receptor, which in a second step facilitates its binding to the CRS2 region (122). This second interaction occurs between the first two N-terminal residues of the ligand and a group of residues mainly found in CRS2 (120). In addition, eight residues in the transmembrane segments TMVI and TMVII link the conformational changes in the transmembrane regions of CXCR4 to the residues involved in signal initiation. This is a critical event for signal transduction, as the residues are part of the microswitch that allows G protein coupling. Notably, residues F248 to V242 in TMVI are in contact with almost all the conserved motifs critical for signaling in GPCRs, including the CWxP motif in TMVI, NPxxY in TMVII, DRY in TMIII, and Y(x)5KL in TMV. These residues play a role in controlling the transition between active and inactive receptor states by allowing helix and side-chain translation, as described in studies using mutational strategies of this hydrophobic bridge in different receptors (123–125). The existence of multiple receptor conformational states, each capable of differentially binding ligand and G protein, suggests the need for a continuum model of ternary complex formation. In addition, several studies have highlighted the role of G protein nucleotide states in the kinetics of ligand binding and receptor conformation (126–128), necessitating the inclusion of G protein activation states in GPCR signaling models. Using a computational approach, a recent report predicted receptor self-associations and designed CXCR4 dimers with different quaternary structures and signaling properties. The authors designed CXCR4 oligomers that activated G_i_, but not all recruited β-arrestins (129) supporting the presence of multiple CXCR4 conformations at the cell membrane.

The ability of GPCRs, including chemokine receptors, to homo- and heterodimerize has been well characterized (130). Evidence from a variety of experimental approaches, including co-immunoprecipitation, cross-linking assays, resonance energy transfer technologies, functional complementation experiments and advanced light microscopy techniques, confirms that GPCRs form both homo- and heterodimers (130). These receptor complexes add additional layers of complexity that also modulate cell responses (131, 132). Crystallographic studies of CXCR4 confirm the existence of dimeric conformations, with the implicated residues mainly located at the extracellular portion of helices V and VI in the case of the CXCR4:IT1t complex, and at the base of helices III and IV in the case of CXCR4:CVX15 (11, 133). However, it is important to note that in these studies, and similarly to the case of the β2-adrenergic receptor (134, 135) and the A2A adenosine receptor (136), the strategy used a T4 lysozyme fusion inserted between TMV and TMVI on the cytoplasmic side of the CXCR4 and a thermostabilizing L125^3.41^W mutation to stabilize the receptor (11, 133). Thus, it was an artifactual strategy to stabilize the conformations, which could influence the results and conclusions obtained. The regions involved in these interactions differ from those previously described in models of GPCR dimerization, where the contacting residues were assigned to helices I and IV (137, 138). The functional implications of these differences for the CXCR4 life cycle remain unclear, but as there is a low sequence identity in the dimerization region between dimerizing GPCRs the data may represent a feature specific to CXCR4.

Chemokine receptor complexes, including CXCR4 and CCR5, appear to form during their synthesis and maturation, and clusters of CXCR4 and CCR5 can be detected in small trans-Golgi vesicles (139). While dimerization may not be required for functional coupling of the GPCR to heterotrimeric G-proteins *per se*, in some cases mutant chemokine receptors that cannot dimerize show a reduced ability to induce cell migration, suggesting that these complexes might be functionally relevant (140, 141). Homo- and heterodimerization processes add complexity to the biology of these receptors and affect their functionality. CXCR4 has been shown to constitutively dimerize (142, 143) and to form heterodimers with other GPCRs, including other chemokine receptors (144–146).

The crystal structure of CXCR4 has shown that the receptor exists as a homodimer (11), suggesting that both wild-type and WHIM mutant forms may coexist as independent monomers, homodimers and/or heterodimers, in cells from patients. *In vitro* studies using FRET and BRET have revealed WHIM homodimers and heterodimerization between some WHIM mutants and CXCR4, providing a molecular mechanism to explain the dominant-negative role of these mutants (77). Although CXCR4 and WHIM alleles are likely to be co-expressed, the stoichiometry of the different complexes could vary between different cell types and patients, potentially contributing to the observed phenotypic heterogeneity of the disease (101). Some recent data in immortalized Jurkat cells expressing CXCR4^R334X^ alone or with wild-type CXCR4 have shown varying levels of impact on signaling cascades (63). Furthermore, the contribution of potential heterodimers between WHIM mutants and other chemokine receptors, including ACKR3, or even with other GPCRs, as demonstrated for CXCR4 (144, 147) may also be relevant in WHIM syndrome and needs to be addressed.

Recent findings using total internal reflection fluorescence microscopy (TIRF-M) have shown that, in addition to the ligand-mediated conformational changes that activate G proteins and influence receptor dimerization, CXCL12 induces CXCR4 nanoclustering. This oligomerization process is associated with conformational changes on the receptor that are required for full activation of the signaling cascade (148, 149). G-protein activation, ERK1/2 and PI3K phosphorylation occur normally in CXCR4 mutants that are unable to nanocluster in the presence of CXCL12 (77, 149) but correct cell polarization, leading edge formation and ligand-mediated directed cell migration (77) require CXCL12-mediated receptor nanoclustering. These findings thus add another layer of complexity because chemokine receptors, like other GPCRs, are dynamic structures embedded in the lipid bilayer of the cell membrane. Receptor nanoclustering is a CXCL12-mediated process that is very limited or absent in unstimulated cells and requires the transduction of conformational changes through the transmembrane helical domains (122), ultimately leading to G protein interaction and signaling. Treatment with pertussis toxin abolishes CXCL12-mediated receptor nanoclustering, suggesting that the process requires ligand binding and receptor activation. Furthermore, the scaffolding role of β-arrestins is critical for proper actin dynamics and receptor nanoclustering, as cells lacking β-arrestin1 have defects in actin dynamics, as well as impaired CXCL12-mediated CXCR4 nanoclustering and weakened cell migration towards CXCL12 gradients (77, 150). Similarly, treatment of CXCR4-expressing cells with latrunculin A, an inhibitor of actin polymerization (149), results in defective CXCL12-mediated nanoclustering and loss of directed cell migration. This evidence thus suggests an active role for the actin cytoskeleton in regulating receptor nanoclustering.

While data are not available for all WHIM CXCR4 mutants, a recent TIRF-M evaluation of the conformation and dynamics of CXCR4^R334X^ at the cell membrane indicates that this mutant is unable to nanocluster in the presence of CXCL12 and blocks CXCR4 nanoclustering when both receptors are co-expressed (heterozygosis). Although CXCR4^R334X^ behaves as a gain-of-function mutant, increasing MAPK and PI3K activation, and promoting stronger chemotaxis when compared with wild-type CXCR4, it fails to promote directed cell migration to CXCL12. Primary T cells expressing CXCR4^R334X^ show CXCL12-mediated polarization but exhibit multiple actin-rich protrusions, suggesting defects in leading edge formation (77). These defects are attributed to insufficient actin cytoskeleton remodeling due to inadequate β-arrestin1 activation when CXCR4^R334X^ is co-expressed. As a consequence, the balance between activated and deactivated cofilin is disrupted and cells fail to reorganize their actin cytoskeleton in the presence of CXCL12. In addition, the receptors exhibit free diffusion at the cell membrane and receptor nanoclustering is lost, consistent with the inability of cells to sense CXCL12 gradients despite the presence of CXCR4^R334X^ homodimers and heterodimers with CXCR4 (77).

Differences in nanoclustering between CXCR4 and CXCR4^R334X^ are unlikely due to the different internal structures of the two receptors. Although it remains to be formally evaluated, we speculate that WHIM mutants and CXCR4 share identical transmembrane structures and the same residues in the cluster that mediate chemokine engagement, signal initiation, propagation and microswitch activation. Differences in the receptors are likely to involve β-arrestin binding and/or activation, which subsequently affect actin cytoskeleton dynamics. For example, although the internal structure of the transmembrane domains is very similar, the absence of 19 residues in the C-terminal end of CXCR4^R334X^ dramatically alters the phosphorylation pattern induced by GRK proteins, which is known to provide a readable barcode for β-arrestin association and function (**Figures 1A, B**) (64). Under such conditions, the association of β-arrestins, as well as the internalization processes, the proper dynamics of the actin cytoskeleton and the ability of the cells to sense the chemoattractant gradient, are altered.

**Figure 3:**
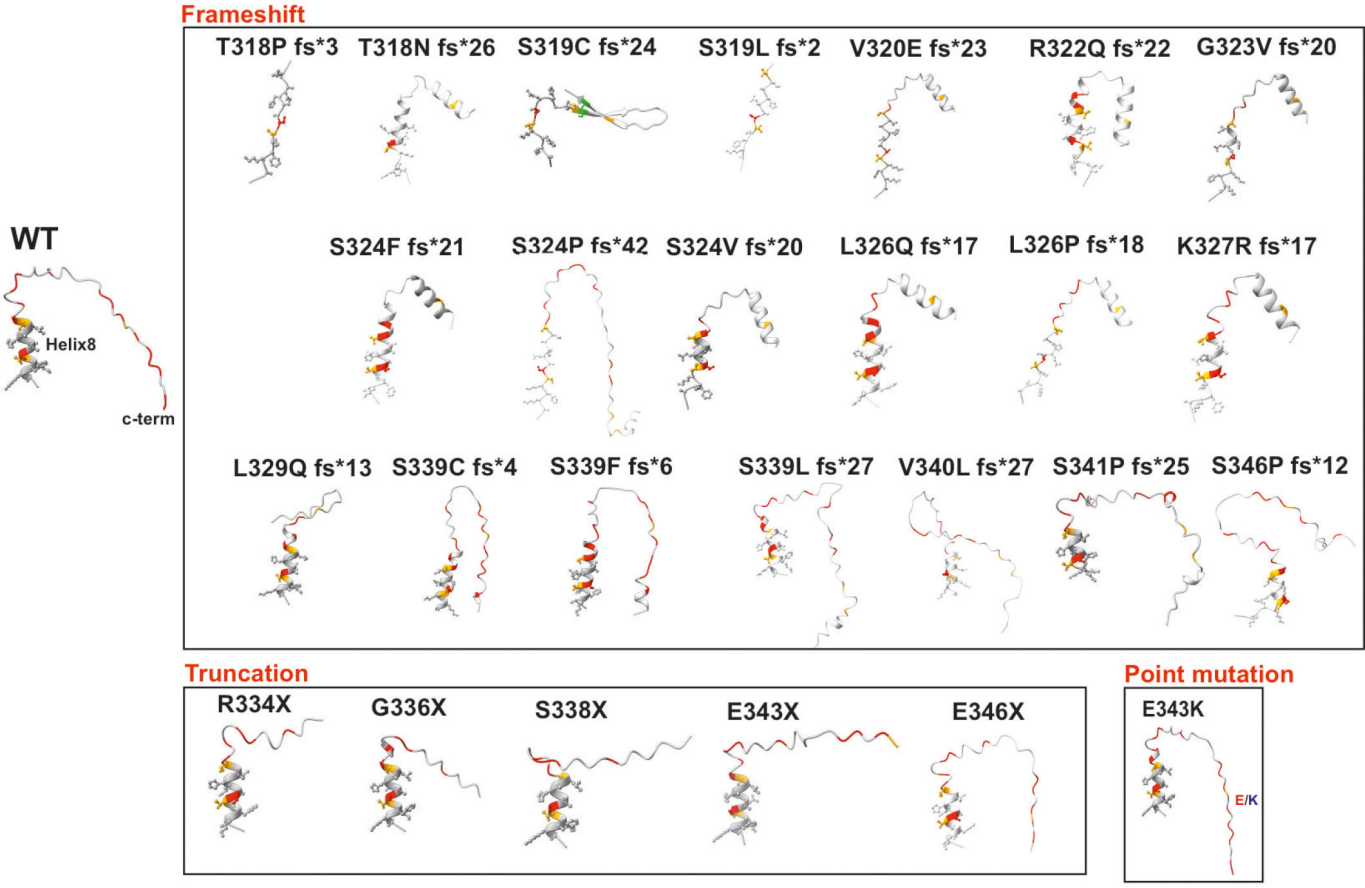
Alphafold2-generated models of helix 8 and C-terminus of wild-type CXCR4 and WHIM syndrome mutants. Models of wild-type CXCR4 helix 8 and the C-terminus end of different mutants associated with WHIM syndrome were generated to analyze the theoretical changes in both the helix and the predicted intrinsically disordered region at the C-terminus. The three different types of mutations present in WHIM syndrome are grouped by black rectangles. The structures are shown as ribbons, with residues belonging to helix 8 shown as spheres and sticks. Serine residues are colored red, threonine residues orange, cysteine residues green and lysine residues in the E343K mutant blue.

From a structural perspective, WHIM mutations may initially compromise the integrity and thus the environment of the intracellular helix 8. This small fragment has been demonstrated to form an amphipathic helix in numerous GPCRs and is critical for stabilizing effective intracellular signaling after ligand binding (151). The generation of helix 8 and C-terminal models for wild-type CXCR4 and WHIM mutants using Alphafold2 (152) has revealed a wealth of different possible conformations and newly acquired secondary structure motifs that may shed light on the impact of a defective C-terminus on CXCR4 function (**Figure 3**). The WHIM mutant models show remarkable differences from the wild-type structure, particularly after a frameshift, where a new sequence is added to the C-terminus. Changes in the integrity of helix 8 can occur, disrupting the folding of the alpha-helix (**Figure 3**), as in the T318P fs*3, L319 fs*24, V320E fs*23 mutants. The addition of new secondary structural motifs may affect both the interaction of the receptor with other proteins and its own oligomerization. In addition, in many of the frameshift mutants, residues that were previously susceptible to phosphorylation either disappeared or shifted their position, altering the pattern of GRK-mediated phosphorylation and thus the barcode used for β-arrestin binding and activation.

## Conclusions

Recent years have seen remarkable progress in understanding the function of chemokine receptors, including receptor structures, interaction with ligand(s), signaling pathways activated and interactions with other membrane proteins. These breakthroughs have defined chemokines as a highly complex family with multiple possible functions depending on the local microenvironment. When analyzing the functionality of WHIM mutants and their association with specific phenotypes in patients, it is crucial to consider the quaternary conformation of the receptors, their interaction with other chemokine receptors (dimers, oligomers) and with other membrane proteins (CD4, tetraspanins, other GPCRs, etc), membrane lipids, and with signaling molecules (G proteins, GRKs, β-arrestins, etc.). Their dysfunction cannot be attributed solely to their lack of internalization and/or degradation.

## Author contributions

JR: Writing – original draft, Writing – review & editing. LG: Writing – review & editing. CS: Writing – original draft, Writing – review & editing. MM: Writing – original draft, Writing – review & editing.
